# Collision risk analysis in the merging area of interchanges using Monte Carlo traffic simulation

**DOI:** 10.1371/journal.pone.0330224

**Published:** 2025-09-15

**Authors:** Dejing Liu, Faxing Li, Zhen Yang, Guilong Xu, Xin Zheng, Song Yue, Yingjun Gao

**Affiliations:** 1 Yunnan Nanjing Highway Co., Ltd., Puer, Yunnan, China; 2 Pu’er Transportation Construction Group Co., Ltd., Puer, Yunnan, China,; 3 The Key Laboratory of Road and Traffic Engineering, Ministry of Education, Tongji University, Shanghai, China; 4 College of Transportation, Tongji University, Shanghai, China; 5 Ningbo Municipal Transportation Bureau, Ningbo, China; 6 National Engineering Research Center for Efficient Maintenance, Safety and Durability of Roads and Bridges, Kunming, Yunnan, China; Southwest Jiaotong University, CHINA

## Abstract

Traffic simulation has gained significant attention due to its ability to quickly evaluate traffic efficiency and safety in the merging areas of interchanges. However, most of the existing studies mainly investigate how to achieve great traffic efficiency, few studies focus on simulation precision and collision risk under traffic uncertainty in merging areas of interchanges. Therefore, the purpose of this paper is to assess potential collision risk in the merging area of interchanges considering uncertainty of traffic flow. A random traffic simulation is established by Monte Carlo method to simulate the high-field traffic flow in the merging area of interchanges. Then the safe braking deceleration (SBD) as a safety measure index is proposed to identify the vehicle’s collision risk in merging. Several risk variables including relative distance, relative speed and merging angular velocity (MAV) between vehicles on the ramp and main line are extracted from the simulation scenario. The results show that the relative speed and distance between the vehicle on the ramp and the one in front on the mainline have little impact on SBD, while MAV significantly indicates collision risk in the merging area of the interchange. Additionally, SBD increases as MAV rises. This study introduces a new traffic simulation platform that accounts for parameter uncertainty to simulate high-fidelity traffic situations, enabling the design of more reliable ramp merging control strategies. Moreover, MAV, which effectively represents SBD, can significantly identify collision risks in the merging area of interchanges.

## 1 Introduction

Mainline merging areas in interchanges play a critical role in merging traffic flow from ramps into the traffic of freeway mainline. In merging areas, vehicles from the ramp are required to enter the mainline through mandatory lane changes within a limited-length lane [1]. leading to traffic disorders for traffic flow in the merging area [2,3]. As a result of this complex environment, merging areas at interchanges are often associated with traffic accidents. Wang, Fu [4] found the accident rate in merging areas of expressway interchanges is 4–6 times of basic road sections. Therefore, investigating drivers’ merging behaviors in merging areas is valuable for reducing accident risk.

Simulation technology offers a convenient and effective method for exploring ways to enhance traffic safety in the merging areas of interchanges [5–7]. Numerous simulation studies have been conducted to optimize lane-changing execution, such as improving merging vehicles’ trajectories and merging orders, to enhance traffic safety [8–12]. For example, according to the results of Essa and Sayed [13] and Hu and Sun [14], vehicle-infrastructure collaboration technology (VICT) can reduce road traffic accident rates by 40% and improve road traffic efficiency by 90% under market penetration rates of 100% connected automated vehicles. In addition, Hou, Zheng [15] developed a cooperative merging control (CMC) model that ensures safe and smooth merging execution for merging vehicles. They found merging and mainline throughput can be promoted by 70% by the CMC strategy with the same safety level of no control.

However, most existing studies on merging areas of interchanges are conducted without accounting for the randomness of traffic flow. In other words, all traffic flow and vehicle parameters are treated as deterministic. This assumption does not align with real-world traffic conditions. For instance, drivers’ reaction times and vehicle lengths are not always the same and may vary across different drivers and vehicles [16–18]. Stochastic traits of driving behavior are also considered as significant factors affecting traffic flow [19–22]. Although numerous studies have been conducted to provide precise traffic flow simulation [23–25], less studies focused on merging areas of interchanges where traffic flow from the mainline interweaves that of ramps. In this complex traffic scenario, stochastic traits of both main line and ramp traffic flow need to be addressed. A simulation that incorporates random traffic characteristics can more accurately simulate real traffic conditions. Hence, there is a need to take these random traffic characteristics into account in the simulation analysis.

According to aforementioned literature review, existing studies aim to improve traffic efficiency through corporative control between mainline and ramp traffic flow. Few studies focus on merging risk in the merging area of interchanges under random traffic simulation. Previous efforts [4,26,27] have indicated higher crash rate in merging areas of interchanges. Traffic safety in random traffic situations needs to be evaluated to understand the impact of traffic randomness on merging risks.

As for assessment of collision risk on merging areas, various safety surrogate measures (SSM) have been proposed in numerous studies [28–31]. These SSM could be divided into four categories: distance-based SSM, time-based SSM, energy-based SSM. The distance-based SSM are indicated by the distance between subject vehicle and leading vehicle. The shorter distance means more dangerous scenario (like space headway). Time-based SSM measure the risk of an interaction in terms of its time proximity to a collision, such as time to collision (TTC), post-encroachment time (PET) [32,33]. Unlike distance-based and time-based SSM, energy-based SSM contemplates severity of potential collisions using collision energy. Examples include Delta-V (∆V) [34] and conflict severity (CS) [35]. Recently, field theory is borrowed from physics to explore risk assessment due to its capability of depicting potential risk source of multiple objectives (e.g., road facilities, vehicles) [36–38]. Arun, Haque [39] also reported a good relationship between risk field and crash frequency and outcome severity. Besides above deterministic methods, probabilistic SSMs considering parameter uncertainty in SSM model have been developed to evaluate safety [40–42]. For example, Shangguan, Fu [43] proposed rear-end crash risk index (RCRI) considering potential crash severity to assess rear-end crash risk, which is calculated by Monte Carlo (MC) simulation. However, MC simulation consumes large computing burden because it samples a lot from random distributions [44], leading to limitation for application. To address this problem, Xu, Yang [45] introduced orthogonal transformation first order reliability method to accelerate computing time of MC, which significantly save the computing resource.

Besides aforementioned SSM, the deceleration-based SSM have often been used in recent years as an evaluation approach for the severity of traffic conflicts [46]. Compared to traditional evaluation methods, the deceleration-based SSM offer distinct advantages. The magnitude of deceleration not only intuitively reflects the abruptness of a vehicle’s speed change but also indirectly indicates the driver’s psychological response to sudden road incidents [47]. If a driver perceives the situation as highly critical, they may slam on the brakes to avoid a collision, resulting in a significant braking deceleration. Conversely, if the driver considers the situation less urgent, they may reduce speed by easing off the accelerator or applying gentle braking, leading to relatively smaller deceleration. In traffic psychology research, it has been found that drivers’ physiological responses differ significantly depending on the magnitude of acceleration or deceleration [48]. Given these advantages of deceleration-based SSM, they could be adopted to appraise risk at merging areas. However, most previous deceleration-based SSM, like Deceleration Rate to Avoid the Crash (DRAC) [49], are introduced without involving driver’s behavior factor (e.g., reaction time). Zheng, Sayed [50] pointed out traffic conflict may evolve towards crash due to driver’s improper behavior. As such, there is needs to proposed new SSM to evaluate crash risk at merging areas of interchanges considering human factors. However, few studies focus on safety assessment using SSM with human behavioral factors.

To address the aforementioned research gaps, a traffic simulation was developed using MATLAB to analyze traffic flow properties. In our custom scenario, traffic parameters in the simulation are discretized using the Monte Carlo (MC) method to model stochastic traffic flow in real-world conditions. A modified car-following model considering Gipps model and driving by visual angle (DVA) model is used to simulate vehicles on the outer lane of main line. An intrusion factor is introduced to capture maximum deceleration rate difference of various drivers. Then the safety braking deceleration (SBD) index is innovatively proposed to identify merging risk. Finally, the influence of relative distance, relative speed and merging angular velocity (MAV) between vehicles on the ramp and main line are analyzed.

By solving exiting problems, this study could reach these contributions: 1. This study supports a more reliable platform to simulate real-word traffic flow using random simulation parameters. 2. A new surrogate safety measure SBD incorporating reaction time of drivers is proposed to evaluate safety at merging area of interchanges. The remaining parts of this study are given as following frameworks: Methodology is shown in **Section** 2. The results and discussion are given in **Section 3**. **Section 4** discusses the potential application of this study, followed by main conclusion of this study in **Section 5**.

## 2 Methodology

To evaluate merging risk between vehicles on the ramp and main line, a simulation framework based on Monte Carlo method was developed. An innovative car-following model that accounts for the intrusion factor, describing the deceleration rate differences between vehicles, was proposed to simulate car-following scenarios. Next, a merging acceleration model incorporating perception coefficient (PC) was used to simulate the merging behavior of vehicles on ramps. Finally, a safety evaluation (SBD) index was utilized to assess merging risk.

### 2.1 Traffic flow on main line

The traffic flow in the interchange merging area consists of vehicles on the mainline and ramps. Assuming that vehicles on the mainline always travel on the outer lane and do not transfer to other lanes. A car-following model considering the intrusion factor was used to simulate the traffic flow on the basis of Gipps model and driving by visual angle (DVA) model. According to the Highway Capacity Manual (HCM2000), we assume the traffic situation is a following state when the headway is less than 5s. Otherwise, it will be in a free flow state.

The Gipps model [51] is one of the most widely used car following models based on safety distance. It comprehensively considers various factors such as vehicle acceleration and deceleration performance limitations, driver compensation response time, and expected speed, and can describe driving behavior in free flow conditions. So inspired by the Gipps model, cars’ acceleration of free flow on main line is given as:


a(t+τ)=2.5anτ(1−vn(t)/vn(t)Vn)(0.025+vn(t)/vn(t)Vn)\raise0.7ex1/12\nulldelimiterspace\lower0.7ex2\nulldelimiterspaceVn)\raise0.7ex1/12\nulldelimiterspace\lower0.7ex2\nulldelimiterspaceVn)(0.025+vn(t)/vn(t)Vn)\raise0.7ex1/12\nulldelimiterspace\lower0.7ex2\nulldelimiterspaceVn)\raise0.7ex1/12\nulldelimiterspace\lower0.7ex2\] (1)

Where a(t+τ) is the acceleration of a following car at time t+τ, m/s²; $a_{n}$ is maximum acceleration of a following car, m/s²; τ denotes reaction time of a following car, s; $v_{n}(t)$ denotes the velocity of a following car at time t, m/s; $V_{n}$ represents the expect velocity of a following car, m/s;

To account for the visual effect of the preceding vehicle’s shade on a following driver’s perception, the driving-by-visual-angle (DVA) model was developed [52]. This model replaces relative velocity and distance parameters with perspective parameters. More specifically, the following vehicle makes acceleration and deceleration decisions based on the perspective and change rate of the preceding vehicle in their view field [53]. As a result of taking into account the driver’s psychological and physiological properties, it can effectively represent the vehicles’ behavior in following flow. Acceleration in following flow is expressed as:


a(t+τ)=0.21(1/1θn(t)−1/1θ¯n(t)\nulldelimiterspaceθ¯n(t)\nulldelimiterspaceθn(t)−1/1θ¯n(t)\nulldelimiterspaceθ¯n(t))−808.25dθn(t)dt (2)

Where $\;\overset{―}{\theta_{n}}(t)$ is the expected visual angle of the following vehicle at time t, rad; $\theta_{n}(t)$ is the visual angle of the following vehicle at time t, rad;

An intrusion factor (IF),${\;C}_{if}$, is introduced to present the maximum deceleration rate difference of various vehicles in an emergency situation. Here we assume the IF has a Gaussian distribution. In traffic simulation, the acceleration in the free flow (formula(1)) and following flow (formula(2)) will multiply $C_{if}$. We can give the final acceleration of the following vehicle:


a(t+τ)={2.5anτ(1−vn(t)/vn(t)Vn)(0.025+vn(t)/vn(t)Vn)\raise0.7ex\(1\)/12\nulldelimiterspace\lower0.7ex\(2\)\nulldelimiterspaceVn)\raise0.7ex\(1\)/12\nulldelimiterspace\lower0.7ex\(2\)\nulldelimiterspaceVn)(0.025+vn(t)/vn(t)Vn)\raise0.7ex\(1\)/12\nulldelimiterspace\lower0.7ex\(2\)\nulldelimiterspaceVn)\raise0.7ex\(1\)/12\nulldelimiterspace\lower0.7ex\(2\)Cif For free flow(0.21(1/1θn(t)−1/1θ¯n(t)\nulldelimiterspaceθ¯n(t)\nulldelimiterspaceθn(t)−1/1θ¯n(t)\nulldelimiterspaceθ¯n(t))−808.25dθn(t)dt)Cif For following flow (3)

### 2.2 Vehicle merging behavior on ramps

The movement of vehicles merging onto the mainline from the ramp’s acceleration lane is more complex than in other road sections. These vehicles must continuously adjust their speed and position to merge seamlessly into the flow of traffic, making a series of acceleration and deceleration decisions based on their relative motion with vehicles on the mainline. If an acceptable gap is available for merging, the driver accelerates and merges directly. If the gap is not suitable, the driver will decelerate to wait for a better gap or the next opportunity to merge.

As such, Sarvi, Ceder [54] developed an acceleration model for merging vehicles and calibrated its parameters using field data. This merging model is adopted in the present study. Contemplating the differences of perceived distance and speed among drivers, relative motion status between merging vehicles and mainline vehicles are multiplied by the driver’s perception coefficient (PC). The acceleration model is given as follows:


$$a_{r}(t+\tau )=-0.134+0.73\left[v_{flead}(t)-v_{r}(t)\right]-0.51\frac{\left[v_{r}(t)-v_{flag}(t)\right]}{\left[x_{r}(t)-x_{flag}(t)\right]}$$ (4)

Where $a_{r}(t+\tau )$ denotes the acceleration of a merging vehicle at time t+τ, $m/s^{2}$; $v_{flead}(t)$ denotes velocity of the leading vehicle on main line, m/s; $v_{flag}(t)$ is velocity of the lag vehicle on main line, m/s; $v_{r}(t)$ is velocity of a merging vehicle, m/s; $x_{r}(t)$ and $x_{flag}(t)$ are the positions of the merging vehicle and lag vehicle respectively.

The traffic flow of the mainline and ramp is simulated. Within each simulation step, the acceleration magnitude of all vehicles is obtained through formulas (3)~(4). Vehicles’ speed and position are also updated accordingly. In addition to updating the position, velocity and acceleration states, merging vehicles will also make lane changing judgments based on the lane changing decision model within each simulation step. If the gap meets the lane changing requirements, merging vehicles will merge into the main line traffic flow. At the same time, the array units of merging vehicles will be initialized in the merging traffic flow and updated in main line traffic flow.

### 2.3 Safe evaluation index

**Fig 1:**
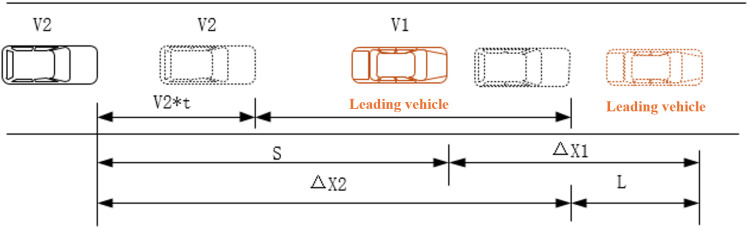
Calculation diagram of Safe Braking Deceleration (SBD).

To assess the potential collision risk during vehicle merging, Safe Braking Deceleration (SBD) is proposed as a safety evaluation index. SBD represents the scenario where the front vehicle exhibits sudden or abnormal driving behavior, while the rear vehicle has no prior warning. The rear vehicle maintains its current speed during the reaction time and continues driving. Then, emergency braking is applied to prevent a collision with the front vehicle, and a minimum safe distance is maintained after both vehicles come to a stop. The minimum deceleration required by the rear vehicle during this process is defined as the SBD, as illustrated in Fig 1:

$\Delta X_{\text{1}}$ represents the travelling distance of the preceding vehicle during the period from decelerating to stopping. $\Delta X_{\text{2}}$ is the traveling distance of the following vehicle, consisting of two parts. First part is the traveling distance during reaction time, and the other is deceleration distance during deceleration time. To avoid collision, the traveling distance difference $\Delta X_{\text{2}}-{\Delta X}_{1}$ should be less than the difference between gap distance before braking and minimum safe distance L. It can be given by following formula:


ΔX2−ΔX1≤S−L⇒V2(τ+tr)−V22/V222a2\nulldelimiterspace2a2+V12/V122a1\nulldelimiterspace2a1≤S−L⇒a2≤V22/V22(2[V2(τ+tr)−(S−L)]+V12/V12a1\nulldelimiterspacea1)\nulldelimiterspace(2[V2(τ+tr)−(S−L)]+V12/V12a1\nulldelimiterspacea1) (5)

Where $a_{2}$ denotes SBD, $m/s^{2}$; $a_{1}$ denotes deceleration of the leading vehicle, $m/s^{2}$; $V_{2}$ and $V_{1}$ is the velocity of the following vehicle and leading one before braking, m/s; $t_{r}$ is reaction lag time of the following vehicle, s. Here it is equal to $\frac{\tau }{2}$. S is gap distance between two vehicles and L is the minimum safe distance.

The Safety Braking Deceleration (SBD) index takes into account multiple factors, such as vehicle speed, distance, acceleration, and the reaction time of different drivers. It also reflects the driver’s physiological state, providing a more accurate representation of collision risk in real-world conditions. The SBD is specifically used to assess the risk posed by a following vehicle on the mainline when a vehicle merges from the ramp.

## 3 Result and discussion

### 3.1 Simulation setting

#### 3.1.1 Road section.

The merging behavior of merging vehicles only interferes with traffic flow of mainline in the vicinity of the merging area, and the impact on mainline traffic flow far from the merging area can be ignored. To accelerate simulation and reduce data storage burden, traffic simulation is set on the road section from 400m upstream of the merging area to 300m downstream of the merging area. The length of the merging area is 300m (the acceleration lane is 230m + the transition section is 70m), and the total length of the road section is 1 km.

According to our field experiment in Yunnan province, most of traffic volume is near 900 veh/h in the mainline, while it ranges between 400 and 500 veh/h at entrance ramps. Therefore, the traffic volume of the outermost lane of the main line is 900 veh/h, and 450 veh/h for the entrance ramp in the proposed simulation. The simulation time interval is set to 0.1s, and the total simulation duration is set to 1000s. A total of 36 simulations were conducted, with a total simulation time of 10 hours.

#### 3.1.2 Traffic flow and driver parameter.

Traffic flow and driver parameters are discretized and randomized using Monte Carlo in micro traffic simulation to simulate real field traffic situations, which includes two parts: traffic flow and drivers’ factors. The first part includes parameters such as time headway, speed distribution, vehicle’s length, expected headway and acceleration fluctuation. The second part involves maximum acceleration, intrusion factor, perception coefficient and driver reaction time.

A shifted negative exponential distribution is adopted to describe the time headway. Due to the small random number generated by the exponential function, which may not be in line with the real following behavior, the minimum headway is set to 1.0s [55]. Statistic information of discrete parameters is given in Table 1.

**Table 1:** Discrete distribution of parameters.

Parameters	Statistic information
Time headway (s)	Shift negative exponential distribution [55]
Initial speed of vehicles on the ramp (m/s)	N~(17, 1.00)
Initial speed of vehicles on the main line (m/s)	N~(23, 1.0)
Expected speed of vehicle(m/s)	N~(25, 2.25)
Maximum acceleration (m/s^2^)	N~(1.7, 0.09)
Length of vehicle (m)	N~(6, 0.09)
Reaction time of driver (s)	N~(1.1, 0.09) [18]
Expected time headway (s)	N~(1.5, 0.04) [56]
Intrusion factor	N~(0.5, 0.0225)
Perception coefficient	N~(1.0, 0.01) [57]
Acceleration fluctuation (m/s^2^)	N~(−0.1, 0.1)

### 3.2 Variables

In the merging area of an interchange, vehicles continuously adjust their motion after entering the acceleration lane from the entrance ramp, while searching for a suitable gap in the mainline traffic to complete the lane change. Due to the limited length of the acceleration lane, the time available for merging vehicles is short, and typically only a few surrounding mainline vehicles directly influence their driving behavior. When merging vehicles first enter the acceleration lane, their speed is usually lower than that of the mainline vehicles, making it difficult for them to overtake and merge into the mainline traffic.

**Fig 2:**
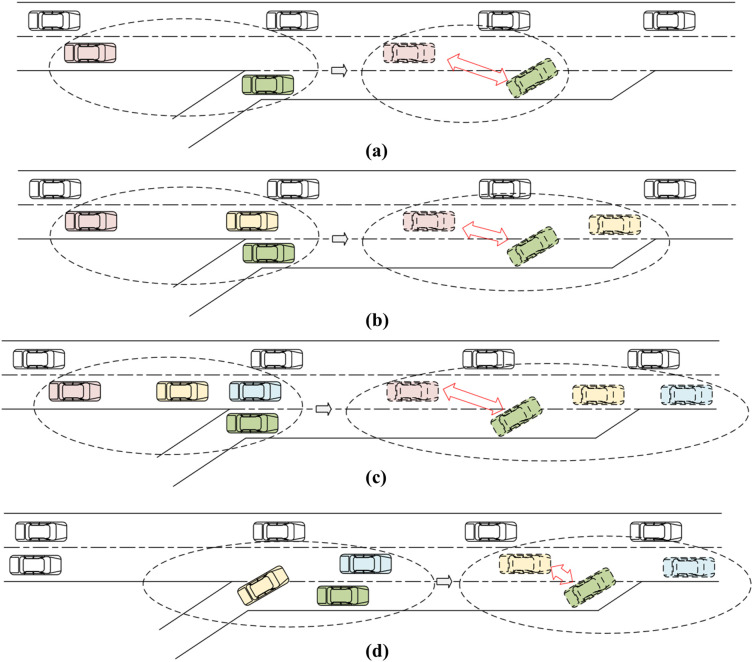
Merging situations: (a) The inserted vehicle is the first vehicle in the main line (b) The inserted vehicle is the second vehicle in the main line (c) The inserted vehicle is the third vehicle in the main line (d) The inserted vehicle is the following vehicle of the merging vehicle.

The situations in which merging vehicles merge into the main line can generally be divided into four types (as shown in Fig 2): a situation where the inserted vehicle is the first vehicle on the main line (Fig 2a); a situation where the inserted vehicle is the second vehicle in the main line (Fig 2b); a situation where the inserted vehicle is the third vehicle behind the main line (Fig 2c) and a situation where the inserted vehicle is the vehicle behind the merging vehicle (Fig 2d). The analyzed objects (vehicles) are connected with the red double-headed arrow, which is in line with modeling of Fig 1. However, this article only analyses the first two situations because statistical results of the simulation test data show that the first and second merging situations of merging vehicles shown in Fig 2a and 2b account for 69.5% and 27.2% of the total number respectively, which means the first and second situations account for 97% of the total, while the third and fourth situation only account for 0.3% and 3% respectively.

In the first situation, the merging vehicle merges into the main lane and cuts in front of the main lane vehicle, making it the first vehicle on the main lane as shown in Fig 2a. In the second situation, the merging vehicle merges into the main lane behind the first main lane vehicle, making it the second vehicle in the main lane as shown in Fig 2b.

A vehicle’s motion state exhibits spatio-temporal continuity, meaning its motion at any given moment evolves from the previous moment. It can be inferred that the risk of vehicle merging in the interchange merging area is closely linked to the relative motion between the merging vehicle and surrounding mainline vehicles when entering the merging area. The simulation test data for the first and second cases are filtered and categorized, and the relative motion between the merging vehicle and the vehicles ahead and behind on the mainline at the moment it enters the acceleration lane is extracted. Additionally, the safe braking deceleration of the mainline vehicle at the moment the merging vehicle merges is analyzed.

**Fig 3:**
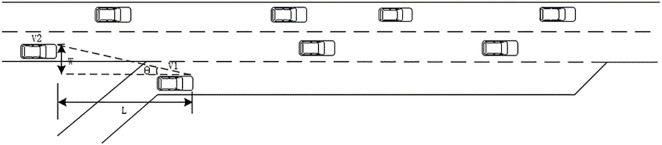
Graphic illustration of Merging Angular Velocity (MAV).


The relationship between SBD and five effect factors are analyzed to explore the collision risk on the merging area. The key elements considered in this article are relative speed between the merging vehicle and the front mainline vehicle ($V_{mergveh}-V_{fvehmainline}$), relative distance between the merging vehicle and the front mainline vehicle ($S_{mergveh}-S_{fvehmainline}$), relative speed between the merging vehicle and the first rear mainline vehicle ($V_{mergveh}-V_{rvehmainline}$), relative distance between the merging vehicle and the rear mainline vehicle ($S_{mergveh}-S_{rvehmainline}$) and Merging Angular Velocity (MAV). The MAV is an integrate indicator contemplating aforementioned relative speed and distance, which is illustrated in Fig 3 and is given as:

dθ/dθdt\nulldelimiterspacedt=−WL2+W2×d(X1−X2)dt=−W(V1−V2)L2+W2≈−W(V1−V2)L2 (6)

Where $X_{1},{\;X}_{2}$ are the locations of vehicle on ramp and mainline respectively, m. $\frac{d\theta }{dt}$ is Merging Angular Velocity (MAV), rad/s; W and L are lateral distance and longitudinal distance between the merging vehicle and the rear mainline vehicle, respectively, m; $V_{1}$ and $V_{2}$ are speeds of vehicles on acceleration lane and main line, respectively, m/s. Eq (6)demonstrates that MAV is a comprehensive metric, which contains relative acceleration and lateral distance between vehicles on ramp and mainline. As such, it can better reflect safety state changes across merging process in spite of low-speed and high-speed situations.

### 3.3 Merging risk analysis

**Fig 4:**
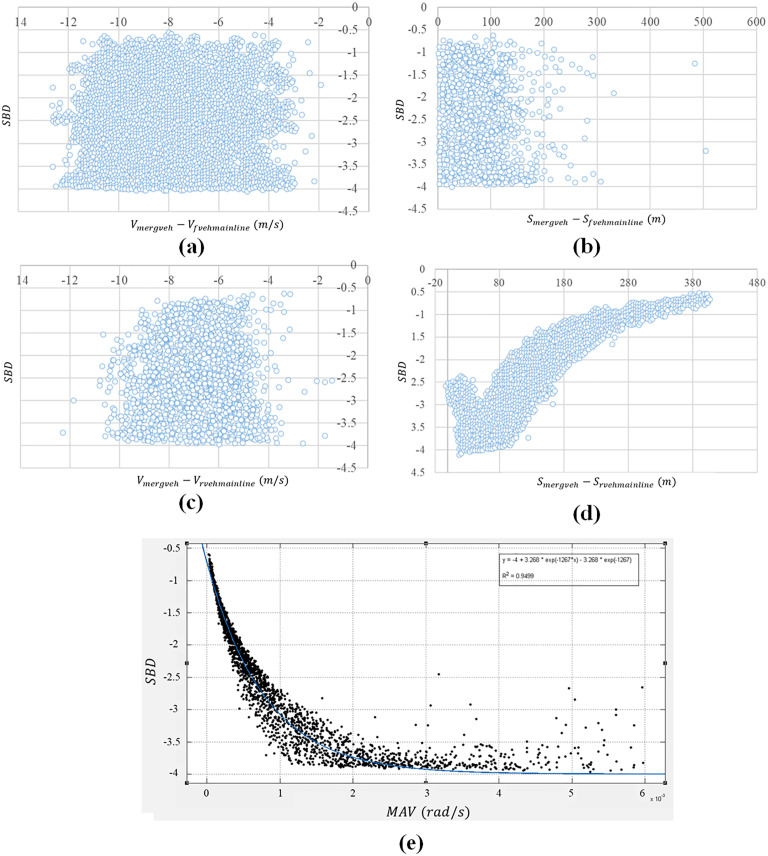
Influence of different variables on SBD in first merging situation.

**Fig 5:**
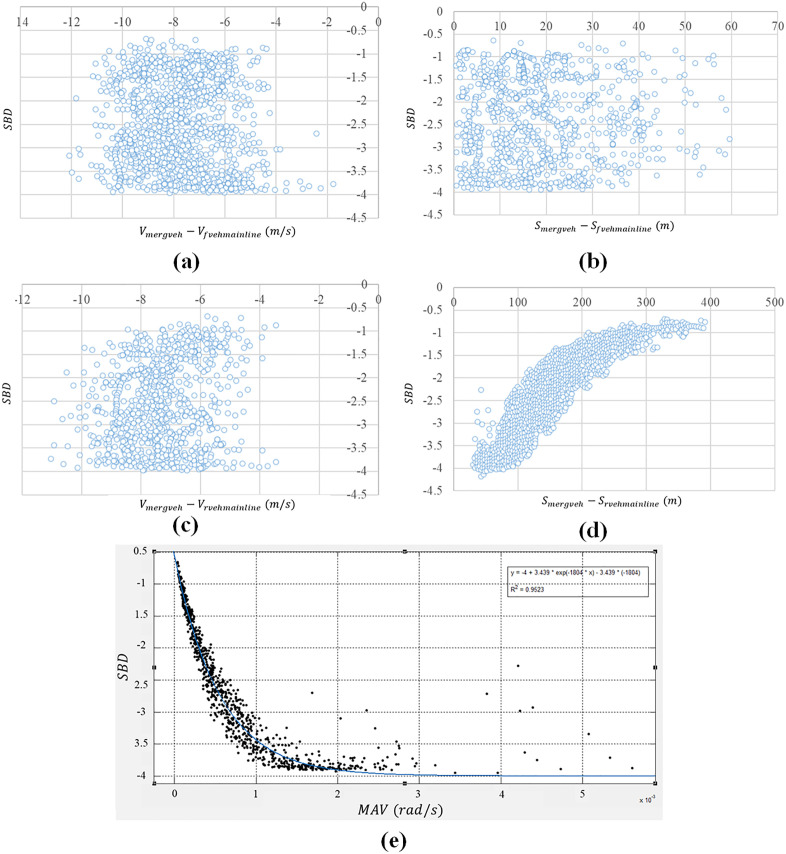
Influence of different variables on SBD in second merging situation.

The SBD and corresponding effect factor data are obtained from proposed Monte Carlo simulation platform. Fig 4 shows the effect of five key factors on SBD in first merging situation (Fig 2a). Statistical description of relative motion near the entrance of interchange is given in Table 2. The counterpart in the second merging situation is also displayed in Fig 5 (Fig 2b). Corresponding statistical information of relative motion is shown in Table 3.

**Table 2:** Statistical description of relative motion in first merging situation.

Variable	Mean	Min	Max	15%	50%	85%
$v_{m}-v_{fv}$	−6.29	−12.16	1.5	−6.6	−3.28	1.5
$s_{m}-s_{f}$	59.10	0.0002	504.38	11.39	47.45	106.06
$v_{m}-v_{r}$	−7.00	−12.27	−0.81	−8.57	−7.00	−5.45
$s_{m}-s_{r}$	111.92	0.14	400.00	48.43	94.91	181.58

Note: $v_{m}$ is speed of merging vehicle on ramp; $v_{f}$ is speed of first vehicle on mainline. $s_{m}$ is distance of merging vehicle on ramp; $s_{f}$ is distance of first vehicle on mainline.$\;v_{r}$ is the speed of rear vehicle on mainline. $s_{r}$ is the distance of rear vehicle on mainline.

**Table 3:** Statistical description of relative motion in first merging situation.

Variable	Mean	Min	Max	15%	50%	85%
$v_{m}-v_{f}$	−7.78	−12.01	−1.75	−9.51	−7.85	−6.03
$s_{m}-s_{f}$	59.10	0.0002	504.38	11.39	47.45	106.06
$v_{m}-v_{r}$	−7.30	−11.00	−3.47	−8.73	−7.32	−5.85
$s_{m}-s_{r}$	131.90	32.84	388.61	68.38	114.79	208.11

Note: $v_{m}$ is speed of merging vehicle on ramp; $v_{f}$ is speed of first vehicle on mainline. $s_{m}$ is distance of merging vehicle on ramp; $s_{f}$ is distance of first vehicle on mainline.$\;v_{r}$ is the speed of rear vehicle on mainline. $s_{r}$ is the distance of rear vehicle on mainline.

First, the statistical description in Tables 2 and 3 reveals that under two different merging scenarios, the relative distance between merging vehicles and mainline vehicles when entering the acceleration lane falls within specific ranges. In the first situation (Table 2), 85% of merging vehicles maintain a relative distance exceeding 48.43 m from the first following vehicle on the mainline. In the second situation (Table 3), 85% of merging vehicles maintain a relative distance exceeding 68.38 m from the second following vehicle on the mainline, while 85% of them keep a distance of less than 31.30 m from the first following vehicle. These findings indicate that when entering the acceleration lane, merging vehicle first assess the relative distance between their vehicle and the mainline following vehicles. If the distance meets the driver’s psychological acceptance threshold, the merging driver will choose to change lanes and merge into the mainline ahead of the first following vehicle. If the distance does not meet the threshold, the merging driver will yield to the first following vehicle and then consider merging ahead of the second following vehicle.

The scatter plots (a) and (b) in both Figs 4 and 5 show that in two different merging situations, the SBD has little correlation with the relative speed and relative distance of the vehicle in front of the merging vehicle. The reason may be that the speed of the front vehicle on the mainline is generally higher than that of merging vehicles, resulting in a lower collision risk between merging vehicles and those on the mainline. Similarly, subplot (c) in Figs 4 and 5 also indicate SBD has less correlation with the speed difference between merging vehicle on ramp and first rear vehicle on main line. The observed phenomenon may be attributed to the fact that when merging vehicles enter the mainline from the acceleration lane, they typically maintain a relatively large distance from the subject mainline vehicle being inserted into [58,59]. Under such conditions, the influence of relative speed on driving behavior becomes negligible compared to that of relative distance. Only when the relative distance decreases sufficiently, the effect of relative speed become noticeably significant.

In Figs 4d and 5d, SBD is significantly influenced by the relative distance between the vehicles on the mainline and those merging. Specifically, SBD decreases as the relative distance increases, with the rate of decrease gradually slowing down, which roughly fits a quadratic curve. However, after the relative distance reaches a certain point, the safety braking deceleration no longer decreases with the increase in relative distance and remains relatively constant. When the merging vehicle enters the acceleration lane and the relative distance between it and the vehicle on the mainline is small, the merging vehicle’s speed is typically lower than that of the mainline vehicle. As a result, at the moment of merging, the relative distance between the merging vehicle and the vehicle on the mainline remains small, and the vehicle on the mainline needs a larger safety braking deceleration to avoid a collision during merging. As the relative distance increases, the required safety braking deceleration for the vehicle on the mainline also decreases. When the relative distance increases to a certain extent, the lane-changing behavior of the merging vehicle has little effect on the vehicle on the mainline, and it no longer significantly impacts the required SBD for the vehicle on the mainline. In the scatter plot (d) of Figs 4 and 5, there is a small portion where the relative distance between the merging vehicle and the vehicle on the mainline is very small, and there is no clear correlation between the safety braking deceleration and the relative distance. This may be due to some aggressive drivers who apply larger acceleration in the acceleration lane. Although the relative distance is small, they still choose to merge in front of the first following vehicle on the mainline.

Compared with the other four elements, there is a high correlation between SBD and MAV in both merging situations. From the scatter plot (e) in Figs 4 and 5, The SBD rises with the increase of the MAV and the magnitude of the increase gradually decreases, roughly fitting an exponential curve. When the MAV exceeds a certain range, the magnitude of the SBD no longer increases with the increase of MAV and remains roughly unchanged.

Low MAV indicates that the relative distance or relative velocity between the inserted vehicle on the main line and the merging vehicle is relatively far or low. When merging vehicles merge into the main line, the relative distance is still relatively large. Therefore, the SBD required for the inserted vehicle on the main line is small. When the MAV is high, it means that the relative distance or relative velocity between the inserted vehicle and the merging vehicle is relatively close or high. When the merging vehicle merges into the main line, two vehicles are in a more dangerous state, demonstrating the inserted vehicle on the main line needs a greater safety braking deceleration to avoid collision. When MAV increases to a certain extent, a slight decrease in relative distance can also lead to a significant increase in MAV, but it basically does not affect the SBD. Therefore, the collision risk basically does not change.

## 4 Potential applications

Proposed traffic simulation model and safety evaluation method could be potentially used in following applications. Firstly, the traffic simulation in this study adopted Monte Carlo (MC) method to simulate real-world traffic flow near merging areas of interchanges. MC method could well reproduce traffic situation in real world because it can cover numerous possible situations in real traffic by randomizing simulation parameters. As such, the model can be employed by transportation agencies to simulate traffic flow in merging areas under various conditions. By incorporating randomness (e.g., variations in driver behavior and vehicle performance), the model can help predict the impact of different traffic management strategies, such as ramp metering, dynamic lane assignments, or signal control. Besides, Researchers and planners can use this simulation tool to test new control systems (e.g., Cooperative Merging Control, vehicle-infrastructure collaboration) and measure their effectiveness in reducing congestion, improving throughput, and minimizing accidents in merging zones.

In addition to traffic simulation, the SBD index can be utilized to assess potential collision risks in merging zones. This allows traffic authorities to identify high-risk areas and take proactive measures to improve safety, such as optimizing lane lengths or adding additional traffic control measures. Safety professionals and researchers can use the new SBD Index to assess risk levels at merging areas in real-time. This tool could be integrated into traffic safety assessment systems to monitor merging areas and identify locations with higher-than-average risk of accidents.

## 5 Conclusion

To assess the collision risk of vehicles in the merging area, a novel traffic simulation platform, discretized and randomized using Monte Carlo methods, is developed. Traffic flow data from the merging area is then collected through the simulation. In this paper, Safe Braking Deceleration (SBD) is proposed as a risk index. Several factors—such as relative distance, relative speed, and MAV between vehicles on the ramp and mainline—are extracted from the simulation scenario. Finally, the impact of these factors on SBD is analyzed, leading to several key findings:

1. The relative speed and distance between a vehicle on the ramp and the vehicle ahead on the mainline have little effect on SBD. This may be because, when a merging vehicle enters the acceleration lane and attempts to merge onto the mainline, the relative distance between the vehicles is typically large. In this case, the impact of relative speed on driving behavior is negligible.
2. The collision risk during merging increases as the relative distance between the vehicle on the ramp and the following vehicle on the mainline grows. However, once this distance exceeds a certain threshold, SBD levels off. Unlike the vehicle ahead, the merging vehicle tends to focus more on the behavior of the vehicle behind it on the mainline when making driving decisions.
3. A strong correlation is found between MAV and SBD, indicating that MAV can be a significant indicator of collision risk in merging areas. As the angular velocity between the merging vehicle on the ramp and the following vehicle on the mainline increases, SBD rises. However, this increase slows down and eventually stabilizes at a low value.

This study introduces a new simulation framework for modeling real-world traffic scenarios in the merging areas of interchanges. SBD is proposed as a risk measure to evaluate collision risk in these areas, providing valuable insights for safety assessments of connected autonomous vehicles in the future. However, further work is required, including the calibration of the traffic simulation model for the merging area and deeper investigation into the relationship between crash data and the SBD index. Besides, MAV and SBD should be verified in more complex situations, like extreme weather condition. Additionally, the influence of random parameters (e.g., Initial vehicle speed, reaction time, perception coefficients) in MC method on simulation results need to be investigated by more strict real-world experiments. Finally, proposed SBD also need to be verified in real-time dataset, like NGSIM, to evaluate its performance.

## Supporting information

S1 Data The .XLSX data file is one part of data produced from simulation platform. (XLSX)

S2 File Note:“vehicle move modify ghr 16.cpp” is the simulation platform programed by c++code,which needs to be loaded by Matlab for traffic flow simulation. (CPP)

## References

[1] Yang H, Ozbay K. Estimation of Traffic Conflict Risk for Merging Vehicles on Highway Merge Section. Transportation Research Record: Journal of the Transportation Research Board. 2011;2236(1):58–65. doi: 10.3141/2236-07

[2] Wan Q, Peng G, Li Z, Inomata FHT. Spatiotemporal trajectory characteristic analysis for traffic state transition prediction near expressway merge bottleneck. Transportation Research Part C: Emerging Technologies. 2020;117:102682. doi: 10.1016/j.trc.2020.102682

[3] Ahammed MA, Hassan Y, Sayed TA. Modeling Driver Behavior and Safety on Freeway Merging Areas. J Transp Eng. 2008;134(9):370–7. doi: 10.1061/(asce)0733-947x(2008)134:9(370

[4] WangX, FuX, GeT. Driving risk evaluation model of freeway interchange entrance area. Journal of Traffic and Transportation Engineering. 2011;11(5):88–92. PubMed

[5] Liu H, Kan X (David), Shladover SE, Lu X-Y, Ferlis RE. Modeling impacts of Cooperative Adaptive Cruise Control on mixed traffic flow in multi-lane freeway facilities. Transportation Research Part C: Emerging Technologies. 2018;95:261–79. doi: 10.1016/j.trc.2018.07.027

[6] Zhu J, Easa S, Gao K. Merging control strategies of connected and autonomous vehicles at freeway on-ramps: a comprehensive review. JICV. 2022;5(2):99–111. doi: 10.1108/jicv-02-2022-0005

[7] Pan T, Guo R, Lam WHK, Zhong R, Wang W, He B. Integrated optimal control strategies for freeway traffic mixed with connected automated vehicles: A model-based reinforcement learning approach. Transportation Research Part C: Emerging Technologies. 2021;123:102987. doi: 10.1016/j.trc.2021.102987

[8] Larsson J, Keskin MF, Peng B, Kulcsár B, Wymeersch H. Pro-social control of connected automated vehicles in mixed-autonomy multi-lane highway traffic. Communications in Transportation Research. 2021;1:100019. doi: 10.1016/j.commtr.2021.100019

[9] Li Y, Li Z, Wang H, Wang W, Xing L. Evaluating the safety impact of adaptive cruise control in traffic oscillations on freeways. Accid Anal Prev. 2017;104:137–45. doi: 10.1016/j.aap.2017.04.025 28500990

[10] Zhibin Li, Pan Liu, Wei Wang, Chengcheng Xu. Development of a Control Strategy of Variable Speed Limits to Reduce Rear-End Collision Risks Near Freeway Recurrent Bottlenecks. IEEE Trans Intell Transport Syst. 2014;15(2):866–77. doi: 10.1109/tits.2013.2293199

[11] Zhang C, Sabar NR, Chung E, Bhaskar A, Guo X. Optimisation of lane-changing advisory at the motorway lane drop bottleneck. Transportation Research Part C: Emerging Technologies. 2019;106:303–16. doi: 10.1016/j.trc.2019.07.016

[12] Xin Z. Real-time warning technology of interchange confluence area based on vehicle-road cooperation. Shanghai: Tongji university; 2018.

[13] Essa M, Sayed T. Self-learning adaptive traffic signal control for real-time safety optimization. Accid Anal Prev. 2020;146:105713. doi: 10.1016/j.aap.2020.105713 32823035

[14] Hu X, Sun J. Trajectory optimization of connected and autonomous vehicles at a multilane freeway merging area. Transportation Research Part C: Emerging Technologies. 2019;101:111–25. doi: 10.1016/j.trc.2019.02.016

[15] Hou K, Zheng F, Liu X, Guo G. Cooperative On-Ramp Merging Control Model for Mixed Traffic on Multi-Lane Freeways. IEEE Trans Intell Transport Syst. 2023;24(10):10774–90. doi: 10.1109/tits.2023.3274586

[16] Kuang Y, Yu Y, Qu X. Novel Crash Surrogate Measure for Freeways. J Transp Eng, Part A: Systems. 2020;146(8). doi: 10.1061/jtepbs.0000405

[17] Kuang Y, Qu X, Wang S. A tree-structured crash surrogate measure for freeways. Accid Anal Prev. 2015;77:137–48. doi: 10.1016/j.aap.2015.02.007 25710638

[18] Xu G, Xu J, editors. Research on deceleration lane length for B-type trumpet interchange. International Conference on Smart Transportation and City Engineering 2021, October 26, 2021 - October 28, 2021; 2021; Chongqing, China: SPIE.

[19] Bouadi M, Jia B, Jiang R, Li X, Gao Z-Y. Stochastic factors and string stability of traffic flow: Analytical investigation and numerical study based on car-following models. Transportation Research Part B: Methodological. 2022;165:96–122. doi: 10.1016/j.trb.2022.09.007

[20] Du J, Jia B, Jiang R, Zheng S-T. Impact of leading speed pattern on oscillation evolution in stochastic linear car-following models. Physica A: Statistical Mechanics and its Applications. 2022;594:127031. doi: 10.1016/j.physa.2022.127031

[21] Wen J, Hong L, Dai M, Xiao X, Wu C. A stochastic model for stop-and-go phenomenon in traffic oscillation: On the prospective of macro and micro traffic flow. Applied Mathematics and Computation. 2023;440:127637. doi: 10.1016/j.amc.2022.127637

[22] XuG, XuJ, ShanH, GaoC, RanJ, MaY, et al. The influence of the pavement friction coefficient evolution caused by traffic flow on the risk of motorway horizontal curves. PLoS One. 2022;17(8):e0266519. doi: 10.1371/journal.pone.0266519 35994492 PMC9394835

[23] Li L, Li S, Gan J, Qu X, Ran B. Revealing the impact of stochastic driving characteristics on car-following behavior with locally collected vehicle trajectory data. Transportmetrica B: Transport Dynamics. 2024;12(1). doi: 10.1080/21680566.2023.2299993

[24] Lee S, Ngoduy D, Keyvan-Ekbatani M. Integrated deep learning and stochastic car-following model for traffic dynamics on multi-lane freeways. Transportation Research Part C: Emerging Technologies. 2019;106:360–77. doi: 10.1016/j.trc.2019.07.023

[25] Xu G, Yang Z, Xie S, Bai S, Liu Z. Enhancing safety and efficiency of signal intersections: A part-time protected right-turn signal control for straight-right lane in connected environment. Physica A: Statistical Mechanics and its Applications. 2025;661:130378. doi: 10.1016/j.physa.2025.130378

[26] Guo Y, Li Z, Liu P, Wu Y. Modeling correlation and heterogeneity in crash rates by collision types using full bayesian random parameters multivariate Tobit model. Accid Anal Prev. 2019;128:164–74. doi: 10.1016/j.aap.2019.04.013 31048116

[27] Song P, Sze NN, Chen S, Labi S. Correcting for endogeneity of crash type in crash injury severity at highway ramp areas. Accid Anal Prev. 2024;208:107785. doi: 10.1016/j.aap.2024.107785 39278137

[28] Wang C, Xie Y, Huang H, Liu P. A review of surrogate safety measures and their applications in connected and automated vehicles safety modeling. Accid Anal Prev. 2021;157:106157. doi: 10.1016/j.aap.2021.106157 33975090

[29] Yuan R, Abdel-Aty M, Zhao Y, Xiang Q. Studying merge behaviour in weaving segments: insights from traffic conflict prediction and risk factors analysis. Transportmetrica A: Transport Science. 2025;:1–26. doi: 10.1080/23249935.2025.2479085

[30] Tang W, Wang H, Ma J, Yang C, Yin C. Vehicle collision risk assessment method in highway work zone based on trajectory data. Traffic Inj Prev. 2025:1–8. doi: 10.1080/15389588.2025.2474722 40138493

[31] Sheikh MS, Peng Y. Modeling collision risk for unsafe lane-changing behavior: A lane-changing risk index approach. Alexandria Engineering Journal. 2024;88:164–81. doi: 10.1016/j.aej.2024.01.028

[32] Hayward JC. Near miss determination through use of a scale of danger. 1972.

[33] Allen BL, Shin BT, Cooper PJ. Analysis of traffic conflicts and collisions. 1978.

[34] Shelby SG. Delta-V as a Measure of Traffic Conflict Severity. 2011.

[35] Bagdadi O. Estimation of the severity of safety critical events. Accident Analysis & Prevention. 2013;50:167–74. doi: 10.1016/j.aap.2012.04.007 22621710

[36] Wang J, Wu J, Li Y. The Driving Safety Field Based on Driver–Vehicle–Road Interactions. IEEE Trans Intell Transport Syst. 2015;16(4):2203–14. doi: 10.1109/tits.2015.2401837

[37] Wang J, Huang H, Li Y, Zhou H, Liu J, Xu Q. Driving risk assessment based on naturalistic driving study and driver attitude questionnaire analysis. Accid Anal Prev. 2020;145:105680. doi: 10.1016/j.aap.2020.105680 32707185

[38] Joo Y-J, Kim E-J, Kim D-K, Park PY. A generalized driving risk assessment on high-speed highways using field theory. Analytic Methods in Accident Research. 2023;40:100303. doi: 10.1016/j.amar.2023.100303

[39] Arun A, Haque MdM, Washington S, Mannering F. A physics-informed road user safety field theory for traffic safety assessments applying artificial intelligence-based video analytics. Analytic Methods in Accident Research. 2023;37:100252. doi: 10.1016/j.amar.2022.100252

[40] Yang Z, Gong Z, Qin Y, Zheng R. Quantifying perceived risk in driving: A Monte Carlo approach for obstacle avoidance. Traffic Inj Prev. 2025;26(3):291–9. doi: 10.1080/15389588.2024.2405647 39417752

[41] Xu G, Xu J, Gao C, Sun R, Shan H, Ma Y, et al. A Novel Safety Assessment Framework for Pavement Friction Evolution Due to Traffic on Horizontal Curves. Sustainability. 2022;14(17):10714. doi: 10.3390/su141710714

[42] Lanzaro G, Alsaleh R, Sayed T. Investigating the impact of correlation on system multimode reliability-based analysis of highway geometric design. Transportmetrica A: Transport Science. 2020;17(4):1027–54. doi: 10.1080/23249935.2020.1826596

[43] Shangguan Q, Fu T, Wang J, Jiang R, Fang S. Quantification of Rear-End Crash Risk and Analysis of Its Influencing Factors Based on a New Surrogate Safety Measure. Journal of Advanced Transportation. 2021;2021:1–15. doi: 10.1155/2021/5551273

[44] Song C, Kawai R. Monte Carlo and variance reduction methods for structural reliability analysis: A comprehensive review. Probabilistic Engineering Mechanics. 2023;73:103479. doi: 10.1016/j.probengmech.2023.103479

[45] Xu G, Yang Z, Ying J, Xie S, Bai S, Qi Y. An integrated potential safety hazard assessment framework in connected car-following scenario. Accid Anal Prev. 2025;216:108010. doi: 10.1016/j.aap.2025.108010 40168704

[46] Fu C, Sayed T. Comparison of threshold determination methods for the deceleration rate to avoid a crash (DRAC)-based crash estimation. Accident Analysis & Prevention. 2021;153:106051. doi: 10.1016/j.aap.2021.106051 33639443

[47] Liu M, Huang XM, Chen YY, editors. Impact elements of roadway traffic on drivers’ mental workload at the highway intersections. 2016 IEEE International Conference on Management of Innovation and Technology (ICMIT); 2016 19-22 Sept. 2016.

[48] Ariën C, Jongen EMM, Brijs K, Brijs T, Daniels S, Wets G. A simulator study on the impact of traffic calming measures in urban areas on driving behavior and workload. Accid Anal Prev. 2013;61:43–53. doi: 10.1016/j.aap.2012.12.044 23477414

[49] Cooper DF, Ferguson N. Traffic studies at T-Junctions. 2. A conflict simulation record. Traffic Engineering & Control. 1976;17(Analytic).

[50] Zheng L, Sayed T, Mannering F. Modeling traffic conflicts for use in road safety analysis: A review of analytic methods and future directions. Analytic Methods in Accident Research. 2021;29:100142. doi: 10.1016/j.amar.2020.100142

[51] Gipps PG. A behavioural car-following model for computer simulation. Transportation Research Part B: Methodological. 1981;15(2):105–11. doi: 10.1016/0191-2615(81)90037-0

[52] Andersen GJ, Sauer CW. Optical information for car following: the driving by visual angle (DVA) model. Hum Factors. 2007;49(5):878–96. doi: 10.1518/001872007X230235 17915604

[53] Saifuzzaman M, Zheng Z. Incorporating human-factors in car-following models: A review of recent developments and research needs. Transportation Research Part C: Emerging Technologies. 2014;48:379–403. doi: 10.1016/j.trc.2014.09.008

[54] Sarvi M, Ceder A, Kuwahara M. Modeling of Freeway Ramp Merging Process Observed During Traffic Congestion. In: Taylor MAP, editor. Transportation and Traffic Theory in the 21st Century: Emerald Group Publishing Limited; 2002. p. 483–502.

[55] Zarean M, Nemeth ZA. WEAVSIM: A microscopic simulation model of freeway weaving sections. 1988.

[56] Aycin MF, Benekohal RF. Linear Acceleration Car-Following Model Development and Validation. Transportation Research Record: Journal of the Transportation Research Board. 1998;1644(1):10–9. doi: 10.3141/1644-02

[57] Hidas P. Modelling lane changing and merging in microscopic traffic simulation. Transportation Research Part C: Emerging Technologies. 2002;10(5–6):351–71. doi: 10.1016/s0968-090x(02)00026-8

[58] Bei R, Du Z, Lyu N, Yu L, Yang Y. Exploring the Mechanism for Increased Risk in Freeway Tunnel Approach Zones: A Perspective on Temporal-spatial Evolution of Driving Predictions, Tasks, and Behaviors. Accident Analysis & Prevention. 2025;211:107914. doi: 10.1016/j.aap.2024.107914 39787825

[59] Wen J, Lyu N, Zheng L. Exploring safety effects on urban expressway diverging areas: crash risk estimation considering extreme conflict types. Int J Inj Contr Saf Promot. 2025;32(1):25–39. doi: 10.1080/17457300.2024.2440940 39676247

